# Characterization, modeling, and anticancer activity of L.arginase production from marine *Bacillus licheniformis* OF2

**DOI:** 10.1186/s12896-024-00829-6

**Published:** 2024-01-25

**Authors:** Manal S. Selim, Marwa M. Mounier, Sayeda A. Abdelhamid, Ahmed Abdelghani Hamed, Mostafa M. Abo Elsoud, Sahar S. Mohamed

**Affiliations:** 1 Microbial Biotechnology Department, National Research Centre, Cairo, Egypt; 2 Pharmacognosy Department, National Research Centre, Cairo, Egypt; 3 Microbial Chemistry Department, National Research Centre, Cairo, Egypt

**Keywords:** Arginase, *Bacillus licheniformis* OF2, Silico homology, Anti-cancer

## Abstract

**Background:**

L-arginase, is a powerful anticancer that hydrolyzes L-arginine to L-ornithine and urea. This enzyme is widely distributed and expressed in organisms like plants, fungi, however very scarce from bacteria. Our study is based on isolating, purifying, and screening the marine bacteria that can produce arginase.

**Results:**

The highest arginase producing bacteria will be identified by using microbiological and molecular biology methods as *Bacillus licheniformis* OF2. Characterization of arginase is the objective of this study. The activity of enzyme was screened, and estimated beside partial sequencing of arginase gene was analyzed. In silico homology modeling was applied to generate the protein's 3D structure, and COACH and COFACTOR were applied to determine the protein's binding sites and biological annotations based on the I-TASSER structure prediction. The purified enzyme was undergone an in vitro anticancer test.

**Conclusions:**

L-arginase demonstrated more strong anti-cancer cells with an IC50 of 21.4 ug/ml in a dose-dependent manner. L-arginase underwent another investigation for its impact on the caspase 7 and BCL2 family of proteins (BCL2, Bax, and Bax/Bcl2). Through cell arrest in the G1/S phase, L-arginase signals the apoptotic cascade, which is supported by a flow cytometry analysis of cell cycle phases.

## Background

Living cells produce enzymes, which are biocatalysts. The catalyst accelerates a chemical reaction [1]. Many environmentally beneficial and profitable industrial industries often use microbial enzymes [2, 3]. The best source of enzymes is microbes because, unlike animal and plant sources, which raise social and political difficulties [4]. A metalloenzyme termed L-Arginase (also known as L-arginine amidohydrolase) catalyzes the L-arginine to L-ornithine and urea [5]. The liver and kidney use the enzyme L-arginase to detoxify ammonia and produce urea, which is used to convert L-ornithine to proline and glutamate [6, 7]. L-arginase has been noticed to have tumour-inhibitory properties and has been given significant consideration because of its wide activity range against cancer cells and to avoid the known risk factors [8–10]. It has been reported to play a crucial role in the treatment of neurological disorders [11], allergic asthma [12], and rheumatoid arthritis [13]. Because argininosuccinate synthetase-1 is not expressed in the same tumors, they are unable to biosynthesize arginine (ASS1). L-arginase primarily controls tumor cell growth and proliferation biochemically through polyamine production [14, 15]. L-arginine depletion via L-arginase is a potent anticancer agent, particularly against malignant melanoma [16] and hepatocellular carcinoma [17]. Contrary to L-arginine restriction's substantial suppression of metastatic expansion, it has been discovered that dietary L-arginine increases the proliferation of tumor cells [17, 18]. L-arginase has been identified from the *Helicobacter pylori* [19], *Neurospora crassa* [20], *Aspergillus nidulans* [21], *Bacillus anthracis* [22], *Bacillus brevis *[23], *Staphylococcus aureus* [24], *Sulfobacillus acidophilus* [25], and mammalian tissues [26]. Arginase seems to have the potential to be used as an imaging biomarker, and [5] examine this possibility in order to generate interest in the creation of increasingly targeted and selective arginase imaging probes. In some of the most well-known arginase-expressing diseases, these imaging probes may become a crucial clinical and scientific tool for estimating the effective concentration of arginase (e.g., immunosuppressive tumors, fibrotic conditions, asthma, atherosclerosis, or carcinomas). This study's objectives were to describe L-arginase from marine bacteria and evaluate these enzymes' in vitro pharmacokinetic properties as anticancer agents against various tumor types of cells.

## Methodology

### Isolation and purification of L-arginase producing bacterial

Marine sediment samples from the Red Sea governorate (Hurghada) for isolation of bacteria. Sediment samples from the shore were collected in sterile tubes and kept in the refrigerator until processed in the laboratory. Nutrient agar medium was used for the isolation and purification of bacteria as described by Suganya et al. [27]. The ingredients were dissolved in 500 ml seawater and the pH was adjusted to 7.0. The final volume was completed up to one liter with distilled water.

### Qualitative screening of L-arginase producing bacteria

Isolates were qualitatively screened for L-arginase activity by streaking on sterilized modified enrichment medium according to Zhang et al. [28] which had the following compositions (g/l): glucose 5.0, arginine 2.5, yeast extract 5.0, peptone 5.0, K_2_HPO_4_ 1.0, agar 20.0, and phenol red reagent added as indicator. The ingredients were dissolved in 500 ml seawater and the pH was adjusted to 7 The final volume was completed up to one liter with distilled water. The inoculated plates were incubated for 48 h at 37ºC. The pink color turned to yellow was positive and were selected for further screening.

### Quantitative screening of L-arginase-producing bacteria

Estimation of L-arginase Activity: The positive isolates were fermented in the media according to Zhang et al.[28] which had the following compositions (g/l): glucose 10, peptone 5, yeast extract 5, K_2_HPO_4_ 1, L-arginine 5, pH 7.0 and the flask were incubated in an incubator shaker at 120 rpm at 37ºC for 48 h. The samples were harvested after 2 days and the cells were separated by centrifugation (5,000 g for 15 min) at 4 °C in a refrigerated centrifuge (SIGMA 3–18 KS). The resultant supernatant was a crude enzyme for enzyme assay and characterization studies.

### Determination of enzyme activity

L-arginase activity was determined based on the amount of urea released in the reaction. Urea on heating reacts with α-isonitrosopropiophenone (Sigma-Aldrich) in the presence of ethanol and produces a pink color which was estimated colorimetrically according to Archibald et al. [29]. The reaction mixture consisted of 0.2 ml of glycine buffer (pH 9.0), 0.5 ml of an enzyme, and 0.1 ml of MnCl_2_. L-arginase was activated by incubating at 37 ºC for 10 min. L-arginine hydrolysis was achieved by incubating the activated arginase with 0.1 ml of L-arginine at 37ºC for 30 min, and 1 ml of per chloric acid was added to arrest the reaction. The urea liberated was estimated by the addition of 0.1 ml of 4% α- isonitrosopriopophenone at 540 nm) using (JASCO V-630) spectrophotometer. Enzyme activity (U/ml) = µmoles of urea released /Time of enzyme action × Volume of the enzyme (ml).

### Soluble protein estimation

Extracellular soluble protein in culture filtrate was estimated by Bradford’s method using bovine serum albumin (BSA) as standard [30].

### Strain identification

Morphological, physiological, and biochemical characterization for the promising bacterium will be carried out. Characteristics of the isolate will be compared with data from Bergey’s manual of determinative bacteriology [31]. The identification will be confirmed with phylogenetic analysis. Genomic DNA from the bacteria was isolated and quality was evaluated on 1.2% agarose gel, a single band of high Mw DNA. The sequence was compared with 16S rRNA sequences in Gen Bank and aligned with close relatives using the BLAST program [32].

### Optimization of the production medium:

#### Optimization of physical parameters for L-arginase production

Optimization of the components of medium required for maximum L- arginase was evaluated. Subsequently, the medium component studied included the effect of different incubation times (24, 48, 72, 96, and 120 h), different pH (6,7, 8, 9, 10, and 11 adjusted with 1 N HCl or 1 N NaOH), different temperatures (25, 30, 35, 40, 45,50, 55, 60 and 65 °C).

#### Optimization of nutritional parameters for L-arginase production

Different additional carbon sources: glucose in the production medium was substituted with other carbon sources, including 1% (w/v) (fructose, sucrose, maltose, and xylose). The various carbon sources were autoclaved separately and added to the medium on an equal carbon basis. Different concentrations of maltose (0.5%, 1%, 1.5%, 2%, and 2.5% w/v) were added. Different nitrogen sources were investigated by substituting the peptone in the production medium, with different sources of nitrogen sources (1%, w/v) (yeast extract, tryptone, and ammonium chloride). Different concentrations of L-arginine (0.5%, 1%, 1.5%, 2% w/v) were substituted in the fermentation medium for the maximum production of L-arginase. Further, the enzyme activity was assayed by Archibald’s method as previously mentioned.

### Purification of L-arginase

Ammonium sulfate (0–80%) saturation was used for protein precipitation according to Dixon, [33]. The most active fraction for L-arginase was centrifuged using SIGMA 3–18 KS (Germany) cooling centrifuge (10,000 rpm,30 min) and the supernatant was dissolved in a minimal amount of 50 mM glycine buffer, pH 9.0, and dialyzed overnight at 4℃ against the same buffer. Then the dialyzate was loaded on a (1.5 X 60 cm) Sephadex G-100 column equilibrated with 50 mM glycine buffer, pH 9, and eluted with one liter of the same buffer at flow rate 0.5 ml/min then collecting eluted fractions (5 ml) for measuring absorbance at 280 nm and the enzyme activity was assayed. The active fractions were pooled and dialyzed against the same buffer and then subjected to a DEAE-cellulose column. Absorbance at 280 nm was measured for the eluted fractions using a UV/VIS-2401 PC spectrophotometer (Shimadzu, Kyoto, Japan) then L-arginase activity and protein content were measured for the most active fractions.

### Characterization of L-arginase

#### Estimation of the molecular weight

Sodium dodecyl sulfate–polyacrylamide gel electrophoresis (SDS-PAGE) was carried out to determine the molecular weight and subunit composition of the enzyme as described by Laemmli, [34]. The molecular weight standards used for SDS-PAGE the gels were stained with 0.25% Coomassie Brilliant BlueR-250.

#### Optimum temperature and thermal stability

The optimum temperature of the purified L-arginase was determined by incubating the reaction mixture at different temperatures ranging from 20–70℃ in 50 mM glycine buffer pH9. The thermal stability of the purified enzyme was determined by pre-incubating the enzyme solution at (10 – 60 min) at various temperatures (from 20 ℃ to 90℃) in the absence of substrate, aliquots were removed and cooled and the residual activity was measured by the standard assay method as mentioned before [1].

#### Optimum pH and pH stability

Purified L-arginase was optimized for its pH using three different buffers with different pH values (6.0–11.0) as follows. Sodium phosphate buffer (50 mM, pH 6.0–7.0), Tris–HCl buffer (50 mM, pH 8.0), glycine buffer (50 mM, pH 9.0), NaHCO_3_–NaOH buffer (50 mM, pH 10.0 and 11) were used to measure the optimal pH for enzyme activity. The relative activities were expressed as a percentage of the maximum enzyme activity. For pH stability measurements, the purified arginase was maintained at pH 6.0–11.0 for 2 h at 4°C then pH values were readjusted to pH 9.0, and then residual enzyme activity was detected by the standard method [35].

### Effect of metal ions

To investigate the influence of metal ions on L-arginase activity, 1 mM final concentration of Na^+^, Ba^2+^, Hg^2+^, Co^2+^, Ca^2+^, Mn^2+^, Mg^2+^, Cd^2+^_,_ and Cu^2+^ was added individually to the reaction mixture at the optimal pH and temperature [36]. Any precipitation that developed was removed by centrifugation. The reaction mixture with no metal ions was used as a control (100% activity).

### Kinetic properties of L-arginase

Kinetic parameters were determined in reaction mixtures containing variable amounts of L-arginine (0.05, 0.1, 0.15, 0.216, 0.25, and 0.3 mM). The Michaelis–Menten constant (Km) and maximum velocity (Vmax) were determined from double reciprocal plots [37].

### Extraction of L-arginase gene

DNA extraction, amplification, and sequencing of the L-arginase gene were carried out using two primers 5’-GGTACCATGGATAAAACGATTTCGG-3’G and 5’-AGCTTTTACAGCAGCTTCT TCCC-3’. Sequencing was carried out at Macrogen, a South Korean public biotechnology company. The amino acid sequence of the arginase was obtained via the translation of the nucleotide sequence of a gene into the amino acid sequence.

### Analysis of physicochemical parameters of L-arginase

The prediction of the secondary structure and determination of the physicochemical parameters of the arginase protein was carried out using ExPASy's ProtParam program. These physicochemical parameters of the arginase protein can be derived from a protein sequence which includes parameters such as molecular weight (M.Wt), instability index (II), aliphatic index (AI), theoretical pI, and grand average of hydropathicity (GRAVY) [38]. the instability index provides an approximation of our protein's stability. A protein with an instability index of less than 40 is projected to be stable; a score greater than 40 indicates that the protein may be unstable [39]**.**

### Construction of the 3D enzymes structure by homology modeling

The amino acid sequences of the arginase enzymes were submitted to the SWISS-MODEL and the 3D structure of the arginase enzymes was automatically generated by first transferring conserved atom coordinates provided by the desired template alignment [40] using formimidoylglutamase from *Bacillus* sp. as a template with (19.25% sequence similarity). The assessment of the predicted 3D structure quality of the homology modeling was carried out via Ramachandran's plot of the model to examine the geometry of residue by residue. The enzyme models were obtained as a PDB file and the model was energy minimized via Gromos96 tools in the Swiss-PDB viewer [41].

### Identification of the enzyme’s catalytic residues

The active-site residues of the arginase enzyme were predicted using the I-TASSER web server (https://zhanggroup.org/I-TASSER/). I-TASSER web server detects catalytic residues in the primary structural alignment, which was then viewed in PyMOL. According to a previously reported approach, the probable active-site residues were superimposed on a template structure in this case [42]. COACH, a meta-server, was then used to predict the protein–ligand interaction site. To construct the final ligand binding site predictions, the predictions were merged with data from the COFACTOR, FIND SITE, and ConCavity analyses.

### Anti-cancer activity

#### Cell lines

Human colorectal carcinoma (HCT-116 cell line), human breast carcinoma (MCF-7 cell line), human prostate cancer (PC3 cell line), human melanoma (Mel501 cell line), human pancreatic tumor cell line (Paca2), human lung carcinoma (A-549 cell line), human melanoma (A-375 cell line), human colon cancer (caco2 cell line), human liver carcinoma (HepG2), and normal human cell line (BJ-1); “a telomerase immortalized normal foreskin fibroblast cell line” were obtained from Karolinska Center, Department of Oncology and Pathology, Karolinska Institute and Hospital, Stockholm, Sweden.

#### Cell culture

The procedure was carried out in a sterile area using a laminar airflow cabinet biosafety class II level. The culture was maintained in RPMI 1640 medium with 1% antibiotic-antimitotic mixture (10,000 U/mL potassium penicillin, 10,000 ug/ml streptomycin sulfate, and 25 ug/ml amphotericin B),1% L-glutamine, and supplemented with 10% heat-inactivated fetal bovine serum. Culturing and sub culturing were carried out according to Thabrew et al. [43]

#### Cell viability assay

This was done according to Mounier et al. [44]. The cells were seeded at concentration of 10 *10^3^cells per well in case of MCF-7 and PC3 and Hep G 2, 20 *10^3^ cells/well in case of A-549, HCT-116, caco2, Mel 501, paca2 and A-375 cell lines and 35–45 *10^3^cells/well in case of BJ-1 using 96-well plates at 37°C. After 48 h of incubation, the medium was aspirated and 40 uL MTT salt (2.5 mg/ml) were added and further incubated for 4 h. Then, 200 ul 10% sodium dodecyl sulphate (SDS) was added. The absorbance was measured at 595 nm.

### Determination of IC_50_ values

IC_50_ values were calculated, using probit analysis, and by utilizing the SPSS computer program (SPSS for windows, statistical analysis software package/version 9/1989 SPSS Inc., Chicago, IL, USA).

### Human CASP-7 (Caspase-7) estimation

The micro ELISA plate provided in this kit is pre-coated with CASP7-specific antibodies. A biotinylated CASP7 antibody and Avidin-Horseradish Peroxidase (HRP) conjugate was added. Aspire the excess components. The substrate solution was added. Wells that contain CASP7, biotinylated detection antibody, and Avidin-HRP conjugate will appear blue. The color turns yellow following the addition of sulphuric acid solution. The optical density (OD) was measured at a wavelength of 450 nm ± 2 nm [45].

#### Measurement of BCl-2 levels

BCL-2 in the samples and standards were estimated according to Barbareschi et al. [46]. A biotin-conjugated antibody was added followed by streptavidin-HRP. The reaction is then terminated by adding acid and absorbance was measured at 450 nm.

#### Measurement of Bax levels

Bax protein levels were evaluated according to Onur et al. [47]. A monoclonal antibody, specific to Bax captured on the plate, was added. After incubation, Streptavidin conjugated to Horseradish peroxidase was added. The reaction was then terminated by adding acid and the optical density of the color produced was measured at 450 nm.

### Cell cycle analysis and apoptosis detection

Cell cycle analysis and apoptosis detection were carried out by flow cytometry. MCF-7 cells were seeded at 1–5 × 10^4^ and incubated at 37 °C, 5% CO_2_ overnight, after treatment with the tested L-arginase, for 24 h, cell pellets were collected and centrifuged (300 × g, 5 min). For cell cycle analysis, cell pellets were fixed with 70% ethanol on ice for 15 min and collected again [48]. The collected pellets were incubated with propidium iodide (PI) staining solution at room temperature for 1 h. Apoptosis detection was performed by Annexin V-FITC apoptosis detection kit (BioVision, Inc, Milpitas, CA, USA) following the manufacturer’s protocol. The samples were analyzed using a FACS Calibur flow cytometer (BD Biosciences, San Jose, CA).

## Results and discussion

### Isolation and screening of arginase producing microbes

**Fig. 1:**
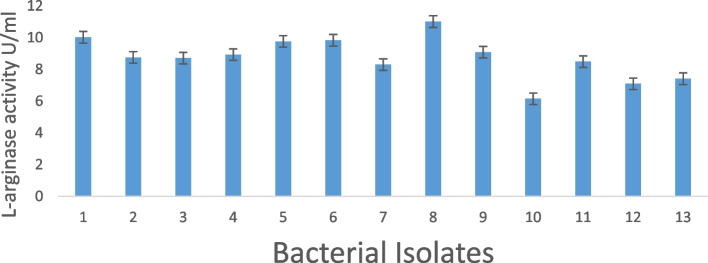
Quantitative L-arginase production from the selected strains

Using a variety of techniques, such as detecting the filtration or color change regions on agar with the completion of the necessary substrate, enzymatic activity was screened [49, 50]. Phenol red displays the fundamental pH change, changing from red in an alkaline environment to yellow in an acidic environment [51]. Thirteen of the 30 marine bacterial isolates that were identified exhibit yellow color surrounding colonies, indicating a drop in pH; these findings are consistent with those made by [52, 53]. Newer strains that can manufacture novel L-arginase had been examined by [54]. It was decided to find and improve the conditions of the highly active bacteria (isolate number 8), which produced L-arginase (11. 01U/ml), as indicated in (Fig. 1).

### Identification of isolate by 16S rRNA gene sequencing

**Fig. 2:**
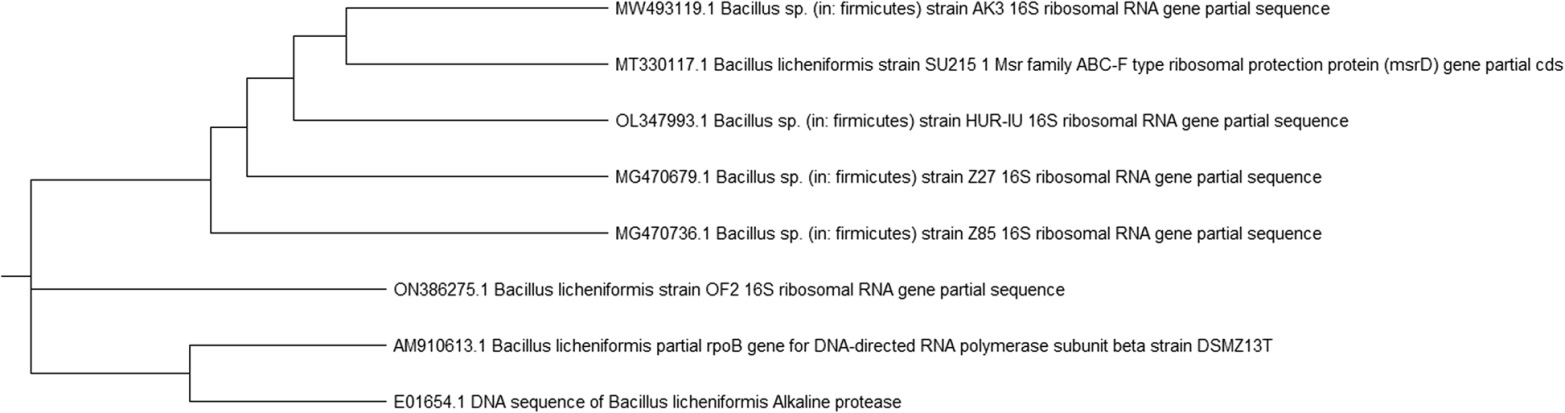
Phylogenetic tree of the *Bacillus licheniformis* OF2 partial 16S rRNA sequence in comparison to closely similar sequences in GenBank databases

Biochemical analyses of the chosen isolate revealed it to be *Bacillus* Sp. Gram-positive, rod-shaped, motile, spore-forming, catalase, urease, nitrate reduction, citrate, Voges-Proskauer, oxidase, 7.5% NaCl, and starch hydrolysis were found to be positive whereas Indole was found to be negative. A molecular method was utilized to confirm and further verify the species identification of the isolate, according to Naveed et al. [55]. The 16S rDNA partial sequence was examined and checked with datasets from Gene Bank. *Bacillus licheniformis* strain OF2, accession number ON386275, was found to be 99% similar to *B. licheniformis*. Based on numerous *Bacillus* species, phylogenetic relationships were created using the neighbor-joining method (Fig. 2).

### Optimization of the production medium:

#### Optimization of physical parameters for L-arginase production

##### Effect of incubation time

**Fig. 3:**
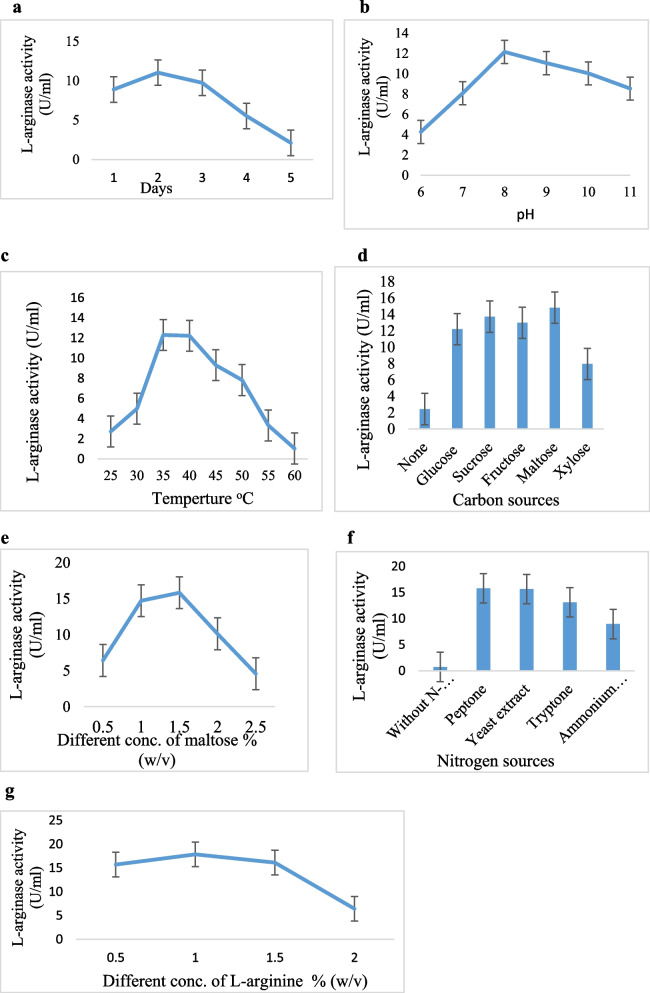
**a** The effect of the incubation time on the production of L-arginase. **b** Effect of the pH on the production of L-arginase from marine *Bacillus licheniformis* OF2. **c** Effect of incubation temperature on L-arginase production from marine *Bacillus licheniformis* OF2. **d** Effect of Carbon sources on L-arginase production from marine *Bacillus licheniformis OF2.* **e** Effect of different concentrations of maltose on L-arginase production from marine *Bacillus licheniformis* OF2. **f** Effect of different nitrogen sources on L-arginase production from marine *Bacillus licheniformis* OF2. **g** Effect of different concentrations of L-arginine on L-arginase production from marine *Bacillus licheniformis* OF2

The duration of the incubation period is essential for the production of enzymes. In the current investigation, L-arginase synthesis began at 24 h, reached a maximum of 48 h (11.03 U/mL), and then reduced with additional incubation (Fig. 3a). Nutritional insufficiency may be the cause of the steady decline in enzyme synthesis [56]. Maximum L-arginase activity was found by Unnisa et al. [57] after 120 h of incubation. The enzyme was produced by *Pseudomonas* sp. strain PV1 after 24 h, according to Nadaf & Vedamurthy, [58].

##### Effect of pH

For maximal microbial growth and a high production of the enzyme, the media should have the ideal pH [59]. Maximum L-arginase activity was seen at pH 8 (12.15 U/m L) (Fig. 3b). When the organism was exposed to a higher pH, the activity then decreased. The findings were confirmed by the findings [54]. As a result, different microbes had varied ideal pH value 8 based on their unique metabolic and nutritional needs. This result contrasted with the arginase from *H*. *pylori*, which, according to research by Zhang et al. [19], preferred an acidic pH and had an optimum pH of 6.1.

##### Effect of temperature

A critical factor in submerged fermentation is temperature. Additionally, due to its impact on the growth of microorganisms and the generation of enzymes, it is a crucial element in every living system [2]. *Bacillus licheniformis* OF2 had the highest L-arginase activity in the current investigation (12.31 U/ml) at 35°C (Fig. 3c). At temperatures above 40°C, L-arginase activity declined. Most likely, a temperature rise may have lowered the number of proteins needed for physiologic and growth activities, which may have prevented bacterial growth [60]. *Alcaligenes faecalis* displayed its highest L-arginase activity at 35°C, according to Ibrahim et al. [61].

#### Optimization of nutritional parameters for L-arginase production

##### Effect of carbon sources

Increased L-arginase production is dependent on the kind and quantity of carbon sources present in the fermentation medium. Maltose (14.82 U/ml) was shown in the current study to be the best source for L-arginase synthesis, followed by sucrose, fructose, glucose, and xylose, in that order (Fig. 3d). According to previous studies, the most efficient carbon source for *Idiomarina sediminum* to produce L-arginase at its highest level is maltose [57]. Maltose was discovered by Elsayed & Danial [62], to be a suitable carbon source for *Bacillus subtilis* to utilize in the production of L-tyrosinase. Glucose and maltose were discovered to be the best sources of carbon for the production of L-glutaminase from the marine *Vibrio costicola* [63]. The maximum activity (15.83 U/ml) was achieved using various doses of maltose, and as the concentrations increased, the activity decreased as shown in (Fig. 3e).

##### Effect of nitrogen sources

In the current study yeast extract, peptone, tryptone, and ammonium chloride were selected as four different nitrogen sources. But as shown mostly by results in (Fig. 3f), the use of peptone (15.78 U/ml) as the nitrogen source resulted in the highest levels of L-arginase activity, which was then followed by the use of yeast extract, tryptone, and ammonium chloride. This might be because it contains complex nutrients such as vitamins, carbohydrates, amino acids, and rich proteins, which could enhance L-arginase activity. In 2020, Nadaf & Vedamurthy [58] reported the same consequence.

#### Different concentration of L-arginine

The amount of L-arginine affects the L-arginase produced. Various L-arginine concentrations (ranging from 0.5 to 2%) were utilized in the current study. Maximum enzyme activity was determined to be produced by 1% of L-arginine (17.84 U/mL) (Fig. 3g). The findings of Nadaf & Vedamurthy [58] were the same. The production of L-arginase is produced by L-arginine, which also serves as a source of carbon and nitrogen. According to Unnisa et al. [57], L-arginine concentration is essential for *Idiomarina sediminum* to produce the most L-arginase.

#### Arginase purification

L-arginase should be purified for its properties to be studied [64]. Table 1 provides an overview of the purification procedures. It was observed that the enzyme's specific activity was determined to be 111.383 U/mg with a yield of 4.522 and fold 5.622% when it precipitated at 40% ammonium sulfate saturation. With a 10.627 fold and 7.132% recovery yield, the partially purified L-arginase was eluted from Sephadex G-100 gel filtration. L-arginase was further purified through loading on DEAE-Cellulose with a purification fold of 23.762, a recovery yield of 4.331%, and a specific activity of 582.42 U/mg protein. These findings are in agreement with those reported by Nakamura et al. [36], who purified L-arginase from *Bacillus subtilis*. Human liver arginase was isolated by Berüter et al. [65] using two chromatography techniques (ion exchange (DEAE-cellulose) and gel filtration) and three precipitation approaches (acetone, heat, and (NH_4_)_2_SO_4_) (Sephadex). Similar findings were also reported by [20, 35, 66].

**Table 1:** Summary of the purification of L-arginase from marine *Bacillus licheniformis* OF2

Purification steps	Total arginase activity (U/ml)	Total protein (mg/ml)	Specific activity (U/mg)	Purification (fold)	Yield (%)
Crude	8875 ± 0.161	362 ± 0.118	24.51 ± 0.154	1	100
(NH_4_)_2_SO_4_ 40%	499 ± 0.091	4.48 ± 0.089	111.383 ± 0.120	4.522	5.622
Sephadex G-100	633 ± 0.115	2.43 ± 0.201	260.49 ± 0.151	10.627	7.132
DEAE-Cellulose	384.4 ± 0.108	0.66 ± 0.096	582.42 ± 0.134	23.762	4.331

#### Characterization of L-arginase

##### Estimation of the molecular weight

An SDS-PAGE evaluation of the final samples revealed that it only contained one band of protein with a molecular weight of 35 k Da. According to [67–69], L-arginase from *Iris hollandica*, *B. licheniformis*, and soybean had 36.5, 33, and 60 k Da, respectively. These findings were in agreement with previous findings. According to [70], variations in the molecular weight of a particular enzyme are caused by the source of the enzyme, the extraction process, and the high accuracy of the purification. Some references also suggest that genetic and environmental situations can have an influence on the molecular weight value.

### Optimum temperature and thermal stability

**Fig. 4:**
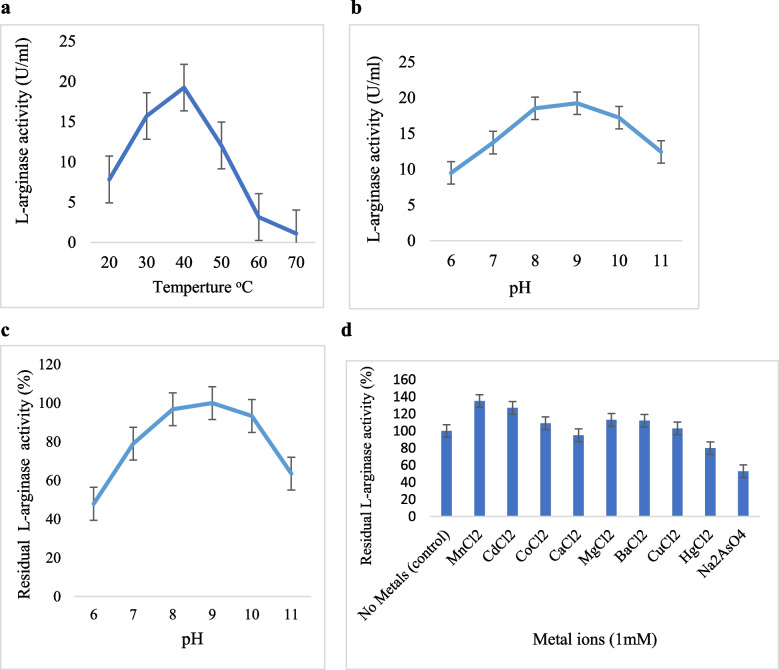
**a** Effect of temperature on the purified L-arginase from marine *Bacillus licheniformis* OF2. **b** Effect of pH on the purified L-arginase from marine *Bacillus licheniformis* OF2 (Sodium phosphate buffer (pH 6 and 7), Tris–HCl buffer (pH 8), glycine buffer (pH 9), and NaHCO_3_–NaOH buffer (pH 10 and 11). **c** pH stability of L-arginase from marine *Bacillus licheniformis* OF2. **d** Effect of metal ions on L-arginase from marine *Bacillus licheniformis* OF2

By measuring the L-activity of L-arginase at varying temperatures, the optimal temperature was obtained. The L-arginase produced in this study was active throughout a wide temperature range (30 to 50°C), with 40°C being the optimum temperature (Fig. 4a). According to Unissa et al. [57], the optimum temperature for the marine bacteria *Idiomarina* sp. and the arginase levels of *Helicobacter pylori* were 37 and 23°C, respectively. *Helix aspersa* and *Helix pomata* revealed optimal temperatures between 60 and 65°C, while arginase from other sources showed comparatively high appropriate temperature values [71].

According to Table 2, L-residual arginase's activity at 20 and 80°C were 100 and 23.31%, respectively. L-thermal arginase's stability investigation revealed that while residual activity was noticeably reduced at temperatures greater than 80°C, this enzyme obtained more than 50% of its activity in the range of 20–70°C. This may be because the enzyme's structure was denatured at the studied temperatures. These findings were in agreement with those of [36, 66]. According to Al-Saad et al. [66], isolated arginase from *Bacillus subtilis* cells did not decrease following a 60-min incubation at temperatures below 55°C.

**Table 2:** Thermal stability of purified L-arginase from marine *Bacillus licheniformis* OF2

Time (min)	Residual activity %					
	**20–40°C**	**50° C**	**60°C**	**70° C**	**80°C**	**90 °C**
**10**	100	95.2	89.7	56.42	23.31	0.30
**20**	100	91.23	82.2	43.1	13.2	0
**30**	100	84.44	75.7	31.6	5.8	0
**40**	100	80.32	70.2	13.5	0	0
**50**	100	74.88	63.1	2.11	0	0
**60**	100	70.13	58.2	0	0	0

- 100% = 19.22 ± 0.101 U/ml

### Optimum pH and pH stability

At an alkaline pH, the purified L-arginase was effective. Maximum activity was reported at pH 9, as shown in (Fig. 4b). but at an acidic pH, activity is reduced. Additionally, as shown in (Fig. 4c)., the residual enzyme activity increased at acidic pH and reduced at alkaline pH. The optimum pH of *Bacillus subtilis* and *Fasciola giant* to produce arginase, which was reported by [72, 73], is between 9.5 and 10. However, according to Viator et al. [22], the pH characteristic of *H. pylori* arginase is distinct at 6.0. The variation in activity with pH suggests that an ionizable group might function at the catalytic site [35].

#### Effect of metal ions

Metal ions may change the charge of the catalytic amino acids and/or cause structural distortions by interfering with the active site at the residues and the enzyme's surface [74]. In this study, the existence of metal ions such as Mn^2+^, Cd^2+^, Ca^2+^, Mg^2+^, Ba^2+^, Cu^2+^ raised the residual arginase activity from 135 to 103%, whereas for cations such as Ca^2+^, Hg^2+^, Na^+^ decreased the residual activity from 95 to 53% (Fig. 4d). A metalloenzyme called L-arginase showed a preference for Mn^2+^, which is similar to the results of previous investigations [6, 35, 72]. According to Nakamura et al. [36], the addition of metal ions to *Bacillus subtilis* arginase increased activation more than other divalent metal ions like Cd^2+^ and Mn^2^ while decreasing activity more than Hg^2+^ and Na^+^. Helicobacter pylori preferred the metals Co^2+^, Ni^2+^, Mn^2+^, respectively [19].

#### Kinetic properties of L-arginase

**Fig. 5:**
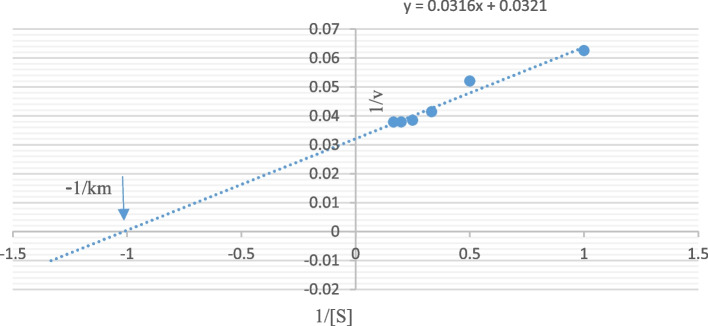
Lineweaver–Burk plot of the purified L-arginase with L-arginine

Figure 5 shows that utilizing arginine as the substrate, the values of K_m_ and V_max_ based on Line Weaver-Burke calculation were 0.112 mM and 36.231 U/ml, respectively. Various L-arginases from microorganisms have different substrate preferences, and they likely perform various physiological functions in the functioning of the enzyme. Higher K_m_ values for L-arginase are found in *F. gigantica*, *Penicillium chrysogenum*, and the marine mollusk *Chitan latus* have higher K_m_ values (6, 4.8, and 25 mM) for l-arginase respectively [72, 75, 76]. On the other hand, L-arginase from *Penicillium americana* used to have a lower km value, which was 0.33 mM [57].

#### Physicochemical properties of L-arginase enzyme

The partial gene sequence of the *B. licheniformis OF2* arginase was obtained and translated to its corresponding amino acid sequence. After obtaining the amino acid sequence, the physicochemical properties of the arginase protein enzyme were predicted using the ProtParam tool. The physical parameter (Table 3) revealed that the L-arginase enzyme had an instability index of (42.19) and a molecular weight of (27,385.13). Arginase is thought to be a thermally stable protein because of its calculated isoelectric point pI value of 5.93 and higher aliphatic index (101.93). The hydrophilicity of the arginase enzyme is shown in Table 3, which indicates its negatively skewed grand average of hydropathicity (GRAVY) values (-0.265). Asp and Glu account for 30 percent of the total negatively charged residues, while Arg and Lys account for 21 percent of the total positively charged residues.

**Table 3:** Summary of the Prot Param data for the arginase

Details	Arginase
Amino acid residue	238
Molecular weight	27,385.13
Theoretical pI	5.93
Positively Charged Residue	21
Negatively Charged Residue	30
Total No. Atoms	3851
Molecular Formula	C_1234_H_1918_N_326_O_367_S_6_
Aliphatic Index (%)	101.93
Instability Index (%)	42.19
GRAVY	-0.265

## Modeling the 3D structures of *L-arginase*

**Fig. 6:**
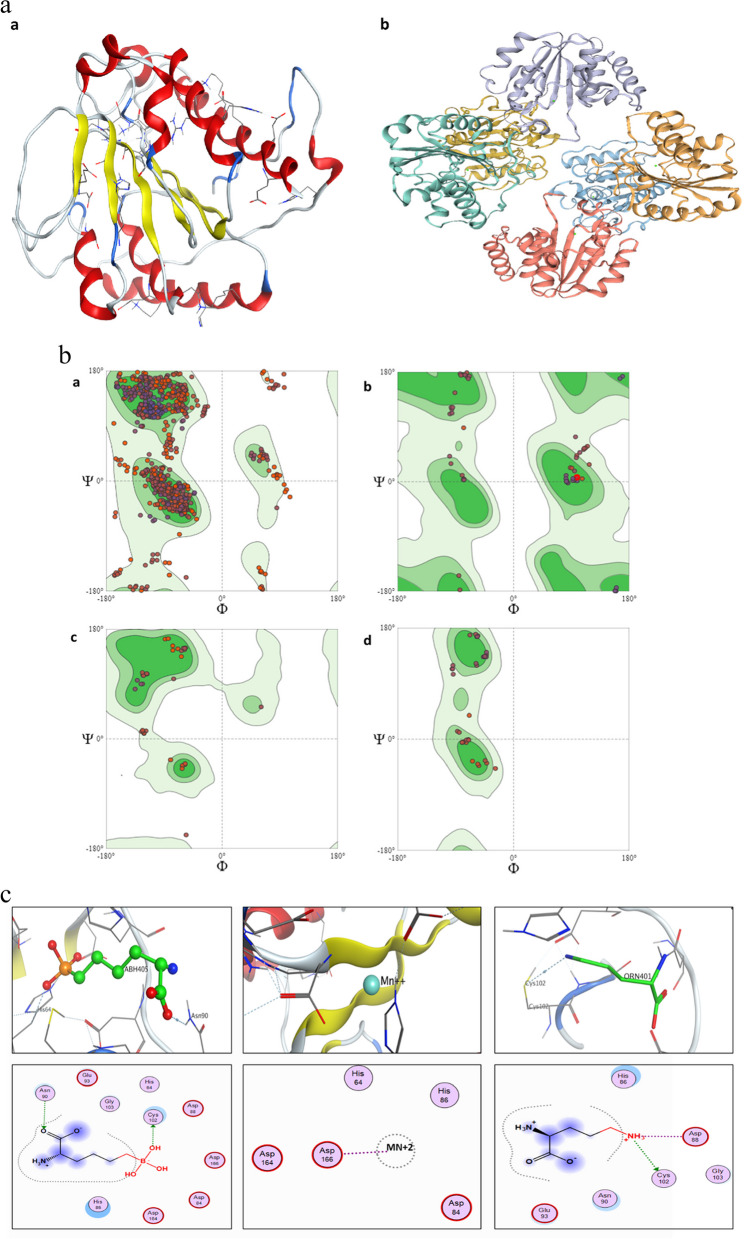
**a** The 3D structures of *Bacillus licheniformis OF2 arginase* enzymes by a) I-Tasser and b) SWISS-MODEL. **b** Ramachandran’s plot calculations on the 3D models of arginase of *Bacillus licheniformis OF2* computed by the SWISS-MODEL web-server to show the favored regions for backbone dihedral angles against amino acid residues in protein structure a) General (No Proline or Glycine) **b**) Glycine Only **c**) Pre-Proline Only **d**) Proline only. **c** predicted ligand binding sites

Homology modeling, also known as comparative modelling, is a computational technique used to predict the three-dimensional structure of a protein based on the known structure of a related protein. This technique is important because it can help us understand the structure–function relationship of a protein, and how it carries out its biological activity. The structure of a protein is crucial for its activity, as it determines how the protein interacts with other molecules in the cell. For example, enzymes require a specific shape to catalyze chemical reactions, and receptors require a specific shape to bind to specific ligands and initiate a signaling cascade. Homology modeling is particularly useful when experimental methods for determining the structure of a protein are difficult or impossible. For example, it may be challenging to determine the structure of a protein that is only expressed in small quantities or is difficult to purify. In these cases, homology modeling can provide a reasonable estimate of the protein's structure, which can be used to study its activity and mechanism of action. In addition, homology modeling is useful for studying the effects of mutations on a protein's structure and function. By predicting the structure of a mutant protein and comparing it to the wild-type protein, we can gain insights into how the mutation affects the protein's activity and mechanism of action. Overall, homology modeling is an important tool for studying the activity and mechanism of action of proteins, particularly when experimental methods for determining the protein's structure are limited [55, 77]. The SWISS-MODEL online server was used to predict the 3D structure of *B. licheniformis* OF2 arginase by homology modeling using the amino acid sequences. *B. licheniformis OF2* arginase enzyme [40]. The Arginase enzyme was constructed using *formimidoylglutamase* from *Bacillus* sp*.* as a template with (19.25% sequence similarity). Figure 6a illustrates the generated 3D structures of *B. licheniformis OF2* arginase prediction by a) I-Tasser and b) SWISS-MODEL.

### Validation of homology modeling

Ramachandran's plot of the model was created to assess the projected 3D structure of the homology modeling and to assess the stereochemical quality of the protein structure by examining the geometry of residue by residue. By calculating the phi (Φ) and psi (ψ) torsion angles, the backbone conformation and overall stereochemical quality of *Bacillus licheniformis* OF2 arginase were determined. The results are shown in the Ramachandran plots in (Fig. 6b).

### Determination of binding site

In silico determination of protein active site using computational methods is important for drug discovery and understanding protein function. The active site is where a ligand, such as a drug molecule, binds to exert its effect, so identifying the active site can aid in identifying potential drug targets and designing drugs that specifically target the active site. Additionally, understanding the active site of a protein is essential for understanding its function, including enzymatic catalysis and binding of specific ligands. In silico determination of the active site can also aid in the study of protein–protein interactions and help elucidate the mechanism of action of a protein. Overall, in silico determination of protein active sites is a valuable tool for drug discovery and the study of protein function [78].

Biological annotations of the obtained protein 3rd structure were assessed via COACH and COFACTOR based on the I-TASSER structure prediction. While COFACTOR predicts the protein functions (ligand-binding sites, EC, and GO) via structure comparison and protein–protein networks, the COACH is a meta-server method that collects various function annotation results (on ligand-binding sites) from the COFACTOR, TM-SITE, and S-SITE programs. According to predictions generated by the I-TASSER algorithm for the 3D structure of a protein, three distinct ligands were assessed for their binding affinity to the protein's binding site. The ligands in question are (S)-2-Amino-6-Boronohexanoic Acid (ABH405) with a predicted binding energy of -9.358 kcal/mol, binding to residues 64, 84, 86, 88, 90, 102, 103, 164, 166, and 207; Manganese (2 +) (MN + 2) with a predicted binding energy of -7.850 kcal/mol, binding to residues 84, 86, 164, and 166; and L-Ornithine (ORN401) with a predicted binding energy of -7.850 kcal/mol, binding to residues 86, 88, 90, 102, and 103 (as depicted in Fig. 6c). These binding energy values reflect the strength of the ligand–protein interactions, with lower values indicating stronger binding. The specific binding site residues listed for each ligand denote the amino acid positions within the protein where binding is anticipated, crucial for forming various types of interactions like hydrogen bonds and hydrophobic contacts. While these predictions offer valuable insights, experimental validation is typically essential to verify their accuracy and biological relevance.

### Biology

The BCL2 family and caspases proteins are key regulating proteins involved in apoptosis. In cancer, the apoptotic pathway is inhibited through up-regulating of different anti-apoptotic proteins and down-regulating of pro-apoptotic proteins leading to intrinsic resistance to the majority of chemotherapeutic anticancer drugs. This makes an urge to search for a new effective anticancer candidate. There are two major apoptotic pathways intrinsic and extrinsic pathways. The extrinsic pathway begins outside the cell when the extracellular environment determines cell death. Unlike the intrinsic pathway, which is called mitochondrial-centred cell death, which is mediated by mitochondrial outer membrane permeabilization (MOMP), The mitochondrial intrinsic pathway is controlled by BCL-2 family proteins, which are bound to the mitochondrial membrane. These proteins act as pro- or anti-apoptotic regulatory proteins [79]. In our study, we aimed to discover a new candidate that signals the intrinsic Mitochondria pathway, so we first examined the L-arginase effect on the mitochondria of nine different tumor cell lines through an MTT- mitochondrial-dependent assay.

### In vitro anticancer activity against human cell lines

**Fig. 7:**
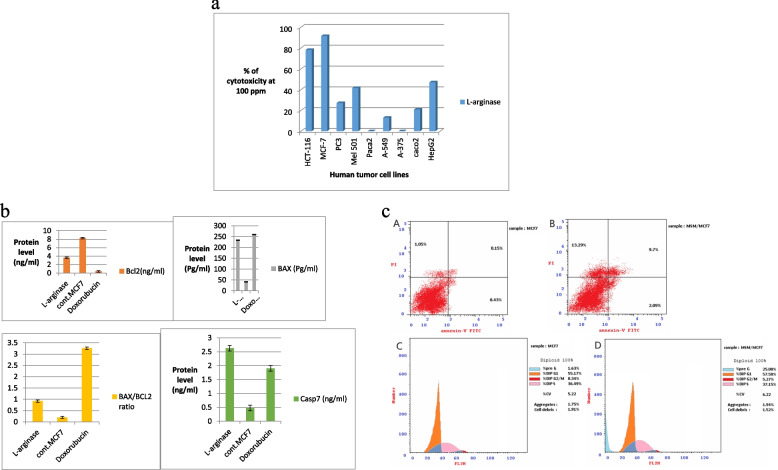
**a** In vitro screening of the antiproliferative activities of L-arginase against human colorectal carcinoma (HCT-116 cell line), human breast carcinoma (MCF-7 cell line), human prostate cancer (PC3 cell line), human melanoma (Mel501 cell line), human pancreatic tumor cell line (Paca2), human lung carcinoma (A-549 cell line), human melanoma (A-375 cell line), human colon cancer (caco2 cell line), human liver carcinoma (HepG2). The preliminary concentration for screening was 100 μg/ml. Each result is a mean of 3 replicate samples and values are represented as % inhibition. **b** BCL2, BAX, BAX/BCL2 ratio and Casp 7 protein level in MCF-7 cells after treatment with L-arginase for 24 h. **c** Cellular mechanism of action of L-arginase (A, B) apoptotic induction of L-arginase. MCF-7 was treated with L-arginase for 24 h and analyzed by annexin V/PI staining. The percentage of apoptotic cells is the sum of early apoptotic (annexin V + /PI −) cell percentage and late apoptotic (annexin V + /PI +) cell percentage. (C, D) Cell cycle analysis of MCF-7 after incubation with compound L-arginase for 24 h. Untreated cells were used as a control

L-arginase has tested its anti-proliferative activity at 100 ug/ml against nine human cancer cell lines namely, human colorectal carcinoma (HCT-116 cell line), human breast carcinoma (MCF-7 cell line), human prostate cancer (PC3 cell line), human melanoma (Mel501 cell line), human pancreatic tumor cell line (Paca2), human lung carcinoma (A-549 cell line), human melanoma (A-375 cell line), human colon cancer (caco2 cell line) and human liver carcinoma (HepG2), as shown in (Fig. 7a).

From above Fig. 7a, L-arginase gave promising activity on breast and colon cell lines, 91.5 and 78.1%, respectively. The Enzyme showed moderate activity at 41.6 and 47.1% on melanoma Mel 501 and liver cell lines, respectively. Unlike L-arginase activity over the rest 5 cell lines remaining, it showed very weak cytotoxic activity ≤ 27.1%. Afterward, the safety and specificity of enzymes were examined against a human normal skin cell line (BJ-1), and according to our results L-arginase, is safe as it possessed minimal cytotoxic activity on BJ-1 (26.1% cytotoxicity) at the same concentration of 100 ug/ml. This encourages us to further screen L-arginase over breast and colon cells at 4 different concentrations (100, 50, 25, and 12.5) ug/ml to calculate their IC_50_ values. The IC_50_ values declare that L-arginase possessed more potency in the breast cell line with IC_50_ 21.4 ± 0.5 than the IC_50_ value on the colon cell line (HCT-116) which was 59.2 ± 0.4. This remarkable response of L-arginase was further elucidated and confirmed by studying the effect of L-arginase on different apoptotic proteins parameter, namely BAX, BCL2, Caspase7, and cell cycle analysis. This study was comparable to that of Niu et al. [80], who investigated the idea that arginase's catalytic enzymatic activity may be used to target arginase enzymes for cancer therapies.

### Cellular mechanism of action

#### Cell apoptosis

##### Effect of L-arginase on the level of BCL2, BAX, BAX/BCL2 ratio and caspase-7 in MCF-7

MCF-7 cells were treated with the IC_50_ of L-arginase (21.4 ug/ml). BCL2 and BAX protein levels were detected in the MCF-7 and compared to untreated MCF-7 cells, as shown in (Fig. 7b). L-arginase downregulates anti-apoptotic protein BCL2 and upregulates pro-apoptotic protein BAX along with its disruption effect of BAX/BCL2 ratio which stimulates apoptotic cascade inside MCF-7 cells, leading to signal the *caspase* 7 which had important roles in mediating cell death signaling. Caspase 7 is the critical mediator of mitochondrial events of apoptosis.

#### Cell cycle arrest

L-arginase induced an increase in early apoptosis compared to untreated cells from 0.43 to 2.09%. Also, the Enzyme signals an increase in late/secondary cellular apoptosis in comparison to untreated MCF-7 cells from 0.15 to 9.7%. Cell cycle distribution by flow cytometry was done to investigate the apoptotic molecular mechanism of L-arginase on breast MCF-7 cells. MCF-7 cells were exposed to L-arginase IC50 (21.4 ug/ml) for 24 h. L-arginase possessed a significant increase in the percentage of cells at the pre-G1 phase by 15.4 folds in comparison to untreated cells. Also, L-arginase increases the accumulation of cells at the S phase by onefold. Our results declared the L-arginase apoptotic cytotoxic effect on breast tumor cells MCF-7 through the cell cycle arrest at the G1/S phase as shown in (Fig. 7c).

## Conclusion

A novel *Bacillus licheniformis* OF2 with the accession number ON386275 demonstrates a higher production of L-arginase in the current study. The activity of the enzyme was screened and estimated, and the partial sequencing of the arginase gene was analyzed. In silico homology modeling was applied to generate the protein's 3D structure, and COACH and COFACTOR were applied to determine the protein's binding sites and biological annotations based on the I-TASSER structure prediction. The purified enzyme underwent an in vitro anticancer test.

## Data Availability

The datasets generated during and/or analyzed during the current study are available from the corresponding author on reasonable request.
